# Proposal of simplified CT syndesmophyte score (sCTSS) and comparison with CTSS in patients with ankylosing spondylitis

**DOI:** 10.1038/s41598-023-28525-z

**Published:** 2023-01-23

**Authors:** Hong Ki Min, Se Hee Kim, Sang-Heon Lee, Hae-Rim Kim

**Affiliations:** 1 Division of Rheumatology, Department of Internal Medicine, Konkuk University Medical Center, 120-1 Neungdong-Ro (Hwayang-Dong), Gwangjin-Gu, Seoul, 143-729 Republic of Korea; 2 Division of Rheumatology, Department of Internal Medicine, Research Institute of Medical Science, Konkuk University School of Medicine, Seoul, Republic of Korea

**Keywords:** Medical research, Rheumatology

## Abstract

The CT syndesmophyte score (CTSS) can evaluate spinal progression more precisely than mSASSS in ankylosing spondylitis (AS); however, it is complex and time consuming. Here, we propose a simplified CTSS (sCTSS) for measuring spinal structural changes in AS. Patients with AS were recruited from a single tertiary hospital. Baseline and 2-year follow-up whole spine CT images were used to calculate CTSS and sCTSS. The sCTSS used the anterior and posterior vertebral corners, and ranged 0–184. Intraclass correlation coefficients (ICC) were calculated, as well as the smallest detectable changes. Fifty AS patients were included. For reader 1, the mean sCTSS at baseline and 2-year follow-up were 11.7 ± 14.6 and 15.8 ± 16.1, whereas those for reader 2 were 12.0 ± 12.5 and 15.8 ± 15.7, respectively. The ICCs for CTSS at baseline and at 2-year follow-up were 0.97 (95% confidence interval [CI] 0.96–0.99) and 0.98 (0.97–0.99), respectively, and that for changes over the 2 years was 0.48 (95% CI 0.23–0.67). For sCTSS, the ICCs were 0.96 (95% CI 0.92–0.97), 0.97 (95% CI 0.94–0.98), and 0.58 (95% CI 0.36–0.74), respectively. Detection rates for syndesmophyte progression were comparable between CTSS and sCTSS. The detection rate for syndesmophytes on only lateral side was 13.2 and 11.4%, and 11.4 and 15.2% at baseline and 2-year follow-up (reader 1 and 2). sCTSS and CTSS showed similar detection rates for syndesmophyte progression. sCTSS may be a reliable method for evaluating spinal structural damage in AS.

## Introduction

Ankylosing spondylitis (AS), a prototype of axial spondyloarthritis, is a type of systemic inflammatory arthritis that triggers arthralgia and stiffness in the axial joints, culminating in inflammation and ankylosis of the spine^1,2^. The treatment goal is to reduce articular symptoms and prevent formation of syndesmophytes in the spine. Therefore, therapeutic efficacy is assessed by measuring disease activity and progression of spinal structural damage. A recent study shows that long-term treatment with tumor necrosis factor inhibitors (TNFi) attenuates spinal structural progression in patients with AS^3^.

The modified Stoke Ankylosing Spondylitis Spinal Score (mSASSS) is the tool used most widely to assess spinal progression in AS^4^. It is calculated from lateral view X-rays of the cervical and lumbar spine^5^. However, the method has several limitations. First, it can be hard to discriminate true structural damage on simple X-rays due to interference from adjacent internal organs and bowel gas. Second, the thoracic spine is not included in the mSASSS; this is important because data from spinal computed tomography (CT) images show that syndesmophytes are most common in the thoracic spine^6^. These critical limitations mean that a novel assessment tool is required for evaluating spinal structural progression in AS, leading to the recent development of the CT syndemophyte score (CTSS)^6^. The CTSS method measure syndesmophytes on cervical, thoracic, and lumbar spine (C2 lower to S1 upper) by subdividing it into four aspects (anterior, posterior, right, and left); in addition, both sagittal and coronal views of each vertebral corner are examined^6^. The CTSS method has definite advantages with respect to evaluation of spinal structural damage in AS, and it can detect spinal progression of AS patients more sensitively than mSASSS^7^. However, the CTSS is time consuming and cannot be scored easily; it is based on a range of 1–4 as follows: 0: no syndesmophytes; 1: syndesmophyte height < 50% of the intervertebral disc space [IDS]; 2: syndesmophyte height > 50% of the IDS; 3: total ankylosis^6^.

Here, we developed a simplified CTSS (sCTSS) for quantifying syndesmophytes in patients with AS. In addition, we compared the ability of the CTSS and sCTSS to detect spinal progression in AS patients.

## Methods

### Patients

Patients with AS were recruited from a single university-based tertiary hospital (Konkuk University Medical Center) from January 2015 to December 2019. The inclusion criteria were as follows: (1) fulfil 1984 modified New York criteria for AS^8^, (2) age > 18, and (3) baseline whole spine CT taken within 12 months of a first diagnosis of AS. Patients with other autoimmune diseases, cancer, pregnancy, or current infection were excluded. The demographic and laboratory data for each patient were collected at the time that the baseline whole spine CT was taken. The whole spine CT was performed to evaluate syndesmophyte progression in AS patients. The study was conducted in accordance with the Declaration of Helsinki (1964 Declaration of Helsinki and its later amendments). Written informed consent was waived by the Institutional Review Board of Konkuk University Medical Center due to the retrospective nature of the study. The study was approved by the Institutional Review Board of Konkuk University Medical Center (approval number: KUMC 2021-05-036).

### Quantification of whole spine CT

Whole spine CT was performed twice per patient: once at baseline and then again 2 years later. Patients were placed in a supine position and scanned using a 64-slice CT scanner (GoldSeal Optima CT 660; GE healthcare, Chicago, IL, USA). A volume CT with 0.625 mm acquisition was acquired from the superior endplate of C1 down to the coccyx (120 kVp, 213 mAs, 110 mm/s, 1.375:1 helical pitch). Next, sagittal and coronal plane views including the complete vertebral column were reconstructed from 2 mm slices. Two independent readers scored the whole spine CT images using the CTSS^6^ and the sCTSS. The CTSS, which ranges from 0 to 552 was calculated as described previously^6^. The sCTSS used only sagittal plane CT images, and divided each vertebral corner into anterior and posterior aspects. The degree of syndesmophyte formation was scored as follows: 0, no syndesmophytes; 1, syndesmophytes, but not bridging; and 2, total ankylosis (bridging). The details are described in Supplementary Table 1, and example of scoring is presented in Supplementary Fig. 1. The CTSS and sCTSS were measured independently by two readers at baseline and at the 2-year follow-up. Both readers were blinded to all patient information and the dates were removed from the scans; the scans were then presented to the readers as Digital Imaging and Communication in Medicine files.

### Statistical analysis and data management

Categorical variables are presented as numbers and percentages. Continuous variables are presented as the median and interquartile range, or as the mean and standard deviation. Inter-reader reliability was measured by calculating the intraclass correlation coefficient (ICC), and Bland–Altman plot. The smallest detectable change (SDC) was calculated using the following equation: SDC = 1.96 × SD_diff_/√k × √2 (SDdiff: standard deviation of the difference of raw progression score between reader 1 and 2, k for number of readers)^6^. The net number of patients with progression above 0, 0.5, or the SDC threshold values was calculated by subtracting the number of patients with a change in the score of < 0, <  − 0.5, or <  − SDC from the number of patients with a change in the score of > 0, > 0.5, or > SDC^6^. *P* values < 0.05 were deemed statistically significant. All analyses were performed using R software (version 3.1.0, R Foundation for Statistical Computing, Vienna, Austria).

### Ethics approval and consent to participate

Written informed consent was waived by the Institutional Review Board of Konkuk University Medical Center due to the retrospective nature of the study. The study was approved by the Institutional Review Board of Konkuk University Medical Center (approval number: KUMC 2021-05-036).

## Results

### Baseline demographics of the enrolled AS patients

Fifty patients with AS were included in the analysis. The median age was 33.5 years, and 38 (76%) were male. Thirty-nine (78%) patients were HLA-B27-positive, and the median erythrocyte sedimentation rate and high sensitivity C-reactive protein level were 8.0 mm/h and 0.1 mg/dL, respectively. Most of the enrolled patients used non-steroidal anti-inflammatory drugs (48/50, 96.0%), and five (10%) were taking a tumor necrosis factor inhibitor. Other characteristics are listed in Table 1.

**Table 1:** Baseline characteristics of the patients with ankylosing spondylitis.

	AS patients
	(N = 50)
Male sex (N, %)	38 (76.0%)
Age (years)	33.5 [28.0–43.0]
Disease duration (years)	0.2 [0.0–1.0]
BMI (kg/m^2^)	23.8 [21.4–25.5]
Laboratory data	
ESR (mm/h)	8.0 [2.0–19.0]
hs-CRP (mg/dL)	0.1 [0.1–0.7]
HLA-B27 positive (N, %)	39 (78%)
Uveitis (N, %)	6 (12.0%)
Psoriasis (N, %)	1 (2.0%)
IBD (N, %)	0
Enthesitis (N, %)	5 (10.0%)
Peripheral arthritis (N, %)	13 (26%)
Dactylitis (N, %)	0
Medication	
NSAID (N, %)	48 (96.0%)
Sulfasalazine (N, %)	34 (68.0%)
Steroid (N, %)	10 (20.0%)
TNFi (N, %)	5 (10.0%)

Variables were presented as the median (interquartile range) or number (percentage).

*AS* ankylosing spondylitis, *BMI* body mass index, *ESR* erythrocyte sedimentation rate, *hs-CRP* highly sensitive C-reactive protein, *HLA* human leukocyte antigen, *IBD* inflammatory bowel disease, *NSAID* non-steroidal anti-inflammatory drug, *TNFi* tumor necrosis factor inhibitor.

### Baseline and 2-year follow-up CTSS and sCTSS, changes in the CTSS and sCTSS over time, and inter-reader reliability of the CTSS and sCTSS

For reader 1, the mean CTSS at baseline and at the 2 year follow-up was 25.7 ± 43.2 and 32.9 ± 46.0, respectively, and the change in the CTSS was 7.2 ± 12.0; for reader 2, these values were 23.0 ± 34.1, 35.0 ± 47.7, and 12.0 ± 16.5, respectively. In the CTSS for reader 1, the lower vertebral corner of T3 and T4 were the most common sites for syndesmophytes at baseline and at 2-year follow-up; the results for reader 2 were similar (Supplementary Fig. 2). The ICC for the CTSS at baseline and at 2-years was 0.97 (95% confidence interval [CI] 0.96–0.99) and 0.98 (95% CI 0.97–0.99), respectively, whereas that for changes in the CTSS was 0.48 (95% CI 0.23–0.67). The CTSS per spinal segment, and the ICC, are summarized in Table 2. For reader 1, the mean sCTSS at baseline and at 2-year follow-up was 11.7 ± 14.6 and 15.8 ± 16.1, respectively, whereas the change in the sCTSS was 4.1 ± 5.9, (similar results were obtained by reader 2). For readers 1 and 2, the most common site of syndesmophytes was the T3 lower border (Supplementary Fig. 3). The ICC for the sCTSS at baseline and at 2-years was 0.96 (95% CI 0.92–0.97) and 0.97 (95% CI 0.94–0.98), respectively, whereas that for changes in the sCTSS was 0.58 (95% CI 0.36–0.74). The mean scores and ICCs per spinal segment are listed in Table 3. The Bland–Altman plots for CTSS and sCTSS were presented in Supplementary Fig. 4.

**Table 2:** Baseline and 2-year follow-up scores, change in the scores, and the ICCs for reader 1 and 2 (CTSS).

Timepoint		Reader 1	Reader 2	ICC (95% CI)
Whole spine (0–552)				
Baseline	Mean (SD)	25.7 ± 43.2	23.0 ± 34.1	0.97 (0.96–0.99)
	Median (IQR)	11.0 [2.0–28.0]	13.0 [3.0–28.0]	
2 years	Mean (SD)	32.9 ± 46.0	35.0 ± 47.7	0.98 (0.97–0.99)
	Median (IQR)	20.5 [6.0–35.0]	20.5 [7.0–42.0]	
Change score (2 years—baseline)	Mean (SD)	7.2 ± 12.0	12.0 ± 16.5	0.48 (0.23–0.67)
	Median (IQR)	5.5 [0.0–11.0]	7.5 [3.0–15.0]	
Cervical spine (0–144)				
Baseline	Mean (SD)	3.2 ± 6.1	2.7 ± 4.0	0.91 (0.85–0.95)
	Median (IQR)	1.0 [0.0–3.0]	1.0 [0.0–3.0]	
2 years	Mean (SD)	5.1 ± 8.5	4.6 ± 7.8	0.96 (0.93–0.98)
	Median (IQR)	2.0 [0.0–5.0]	2.0 [1.0–4.0]	
Change in score (2 years—baseline)	Mean (SD)	1.9 ± 4.0	1.9 ± 4.3	0.69 (0.51–0.81)
	Median (IQR)	1.0 [0.0–2.0]	1.0 [0.0–3.0]	
Thoracic spine (0–264)				
Baseline	Mean (SD)	16.3 ± 30.3	13.9 ± 22.5	0.97 (0.95–0.98)
	Median (IQR)	7.0 [1.0–17.0]	7.0 [2.0–17.0]	
2 years	Mean (SD)	19.8 ± 31.4	21.3 ± 32.1	0.98 (0.96–0.99)
	Median (IQR)	12.5 [2.0–21.0]	11.0 [5.0–26.0]	
Change score (2 years—baseline)	Mean (SD)	3.4 ± 9.9	7.3 ± 12.2	0.46 (0.20–0.65)
	Median (IQR)	2.0 [0.0–6.0]	5.0 [2.0–9.0]	
Lumbar spine (0–144)				
Baseline	Mean (SD)	6.2 ± 10.9	6.3 ± 10.5	0.95 (0.92–0.97)
	Median (IQR)	1.5 [0.0–7.0]	2.0 [0.0–8.0]	
2 years	Mean (SD)	8.0 ± 11.3	9.1 ± 12.8	0.97 (0.95–0.98)
	Median (IQR)	4.0 [1.0–9.0]	3.5 [1.0–13.0]	
Change score (2 years—baseline)	Mean (SD)	1.9 ± 2.5	2.8 ± 3.9	0.36 (0.11–0.58)
	Median (IQR)	1.0 [0.0–3.0]	2.0 [0.0–5.0]

**Table 3:** Baseline and 2-year follow-up scores, changes in the scores, and the ICC between reader 1 and 2 (sCTSS).

Timepoint		Reader 1	Reader 2	ICC (95% CI)
Whole spine (0–184)				
Baseline	Mean (SD)	11.7 ± 14.6	12.0 ± 12.5	0.96 (0.92–0.97)
	Median (IQR)	7.0 [2.0–17.0]	8.5 [3.0–16.0]	
2 years	Mean (SD)	15.8 ± 16.1	15.8 ± 15.7	0.97 (0.94–0.98)
	Median (IQR)	11.5 [5.0–22.0]	12.5 [6.0–20.0]	
Change score (2 years—baseline)	Mean (SD)	4.1 ± 5.9	3.8 ± 6.7	0.58 (0.36–0.74)
	Median (IQR)	4.0 [0.0–7.0]	3.0 [0.0–5.0]	
Cervical spine (0–48)				
Baseline	Mean (SD)	1.6 ± 3.0	1.7 ± 2.3	0.87 (0.78–0.93)
	Median (IQR)	0.5 [0.0–2.0]	1.0 [0.0–2.0]	
2 years	Mean (SD)	2.8 ± 3.8	2.5 ± 3.6	0.90 (0.82–0.94)
	Median (IQR)	1.5 [0.04.0]	1.0 [0.0–3.0]	
Change score (2 years—baseline)	Mean (SD)	1.1 ± 2.0	0.7 ± 2.1	0.40 (0.14–0.61)
	Median (IQR)	0.5 [0.0–2.0]	0.0 [-1.0–1.0]	
Thoracic spine (0–88)				
Baseline	Mean (SD)	7.1 ± 9.8	7.0 ± 8.2	0.94 (0.90–0.97)
	Median (IQR)	3.0 [1.0–11.0]	4.0 [1.0–11.0]	
2 years	Mean (SD)	9.2 ± 10.6	9.2 ± 10.8	0.95 (0.92–0.97)
	Median (IQR)	5.0 [1.0–14.0]	6.0 [3.0–15.0]	
Change score (2 years—baseline)	Mean (SD)	2.1 ± 4.8	2.2 ± 5.3	0.51 (0.27–0.69)
	Median (IQR)	1.0 [0.0–4.0]	1.0 [0.0–4.0]	
Lumbar spine (0–48)				
Baseline	Mean (SD)	3.0 ± 4.2	3.3 ± 4.1	0.95 (0.92–0.97)
	Median (IQR)	1.0 [0.0–5.0]	2.0 [0.0–5.0]	
2 years	Mean (SD)	3.9 ± 4.2	4.1 ± 4.1	0.93 (0.87–0.96)
	Median (IQR)	3.0 [1.0–5.0]	3.0 [1.0–6.0]	
Change score (2 years—baseline)	Mean (SD)	0.9 ± 1.8	0.9 ± 2.1	0.55 (0.32–0.72)
	Median (IQR)	1.0 [0.0–2.0]	1.0 [0.0–2.0]	

### Detection of syndesmophytes by the CTSS and sCTSS

**Figure 1:**
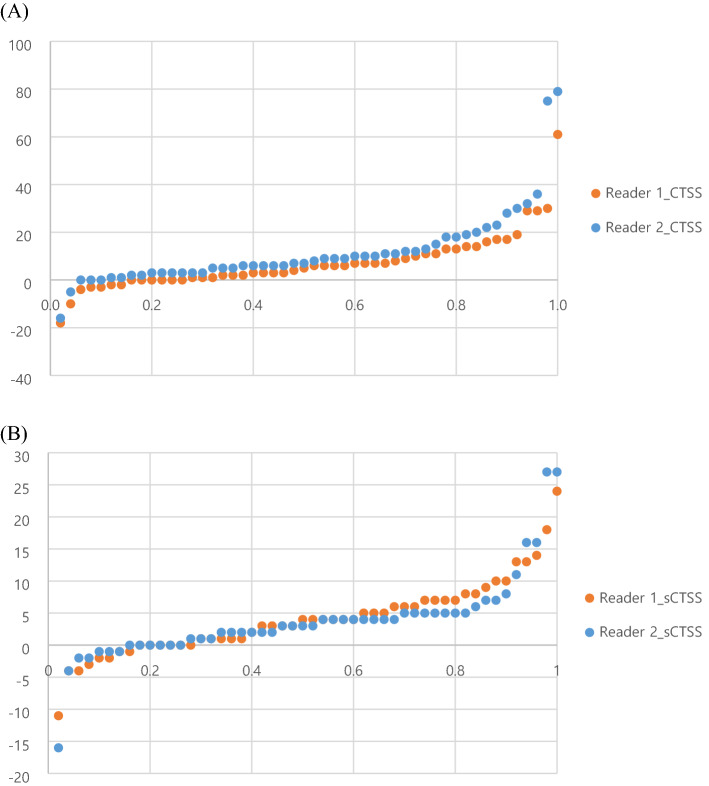
Cumulative probability plot of 2 year progression scored by reader 1 and 2 in (**A**) CTSS and (**B**) sCTSS methods.

The cumulative probability plots for CTSS and sCTSS for both readers were presented in Fig. 1. Next, we compared the ability of the two scores to detect syndesmophyte progression. We did this by setting cut-off values of 0, 0.5, and SDC. The SDC of the whole spine was 14.7 for CTSS and 5.7 for sCTSS. For the CTSS, the detection rates for net progression (i.e., subtracting the number of negative changes from the number of positive changes) were 7/50 (14%) and 12/50 (24%) for reader 1 and 2, respectively, when setting the SDC as the cut-off value for determining progression. For the sCTSS, these rates were 16/50 (32%) and 8/50 (16%) for reader 1 and 2, respectively. For readers 1 and 2, the ability of the CTSS and sCTSS to detect spinal progression was comparable (*p* = 0.07 and *p* = 0.47, respectively). There was no significant difference between the CTSS and the sCTSS with respect to the ability to detect meaningful progression when using cut-off values of 0 nor 0.5. The detailed SDCs and detection rates per spinal segment are described in Table 4. When counting sites of new syndesmophytes, or syndesmophyte growth (as described in Supplementary Table 1), readers 1 and 2 identified the T3 lower vertebral corner as the most common site in the CTSS; this site was also identified by reader 2 using the sCTSS. Except for sCTSS in reader 1, lower vertebral corner of C5 was the most frequently new syndesmophyte or syndesmophyte growth were found (Supplementary Fig. 5).

**Table 4:** Number of AS patients with progression, as determined by the CTSS and sCTSS.

			CTSS Reader 1 n (%)	CTSS Reader 2 n (%)		sCTSS Reader 1 n (%)	sCTSS Reader 2 n (%)	*P* for reader 1*	*P* for reader 2*
	SDC				SDC				
Whole spine									
Change > 0.0		Positive	37 (74)	45 (90)		36 (72)	37 (74)	0.99	0.07
		Negative	7 (14)	2 (4)		8 (16)	7 (14)		
		Net	30 (60)	43 (86)		28 (56)	30 (60)		
Change > 0.5		Positive	31 (62)	41 (82)		31 (62)	34 (68)	0.99	0.17
		Negative	5 (10)	2 (4)		6 (12)	4 (8)		
		Net	26 (52)	39 (78)		25 (50)	30 (60)		
Change > SDC	14.7	Positive	8 (16)	13 (26)	5.7	17 (34)	9 (18)	0.07	0.47
		Negative	1 (2)	1 (2)		1 (2)	1 (2)		
		Net	7 (14)	12 (24)		16 (32)	8 (16)		
Cervical spine									
Change > 0.0		Positive	30 (60)	28 (56)		25 (50)	23 (46)	0.42	0.42
		Negative	4 (8)	9 (18)		4 (8)	13 (26)		
		Net	26 (52)	19 (38)		21 (42)	10 (20)		
Change > 0.5		Positive	11 (22)	13 (26)		15 (30)	12 (24)	0.49	0.99
		Negative	2 (4)	1 (2)		1 (2)	1 (2)		
		Net	9 (18)	12 (24)		14 (28)	11 (22)		
Change > SDC	3.2	Positive	8 (16)	8 (16)	2.2	8 (16)	9 (18)	0.99	0.99
		Negative	1 (2)	1 (2)		1 (2)	1 (2)		
		Net	7 (14)	7 (14)		7 (14)	8 (16)		
Thoracic spine									
Change > 0.0		Positive	30 (60)	40 (80)		30 (60)	30 (60)	0.99	0.06
		Negative	12 (24)	5 (10)		8 (16)	9 (18)		
		Net	18 (36)	35 (70)		22 (44)	21 (42)		
Change > 0.5		Positive	23 (46)	31 (62)		23 (46)	23 (46)	0.99	0.16
		Negative	6 (12)	2 (4)		7 (14)	6 (12)		
		Net	17 (34)	29 (58)		16 (32)	17 (34)		
Change > SDC	11.4	Positive	5 (10)	8 (16)	4.9	12 (24)	8 (16)	0.11	0.99
		Negative	2 (4)	0		2 (4)	1 (2)		
		Net	3 (6)	8 (16)		10 (20)	7 (14)		
Lumbar spine									
Change > 0.0		Positive	34 (68)	34 (68)		28 (56)	28 (56)	0.30	0.30
		Negative	4 (8)	4 (8)		7 (14)	8 (16)		
		Net	30 (60)	30 (60)		21 (42)	20 (40)		
Change > 0.5		Positive	19 (38)	20 (40)		14 (28)	17 (34)	0.40	0.68
		Negative	2 (4)	1 (2)		3 (6)	4 (8)		
		Net	17 (34)	19 (38)		11 (22)	13 (26)		
Change > SDC	3.7	Positive	9 (18)	16 (32)	1.8	14 (28)	17 (34)	0.34	0.99
		Negative	0	1 (2)		3 (6)	4 (8)		
		Net	9 (18)	15 (30)		11 (22)	13 (26)		

*P values, detection of positive progression by the CTSS versus the sCTSS.

### Syndesmophytes found only on coronal sections

**Figure 2:**
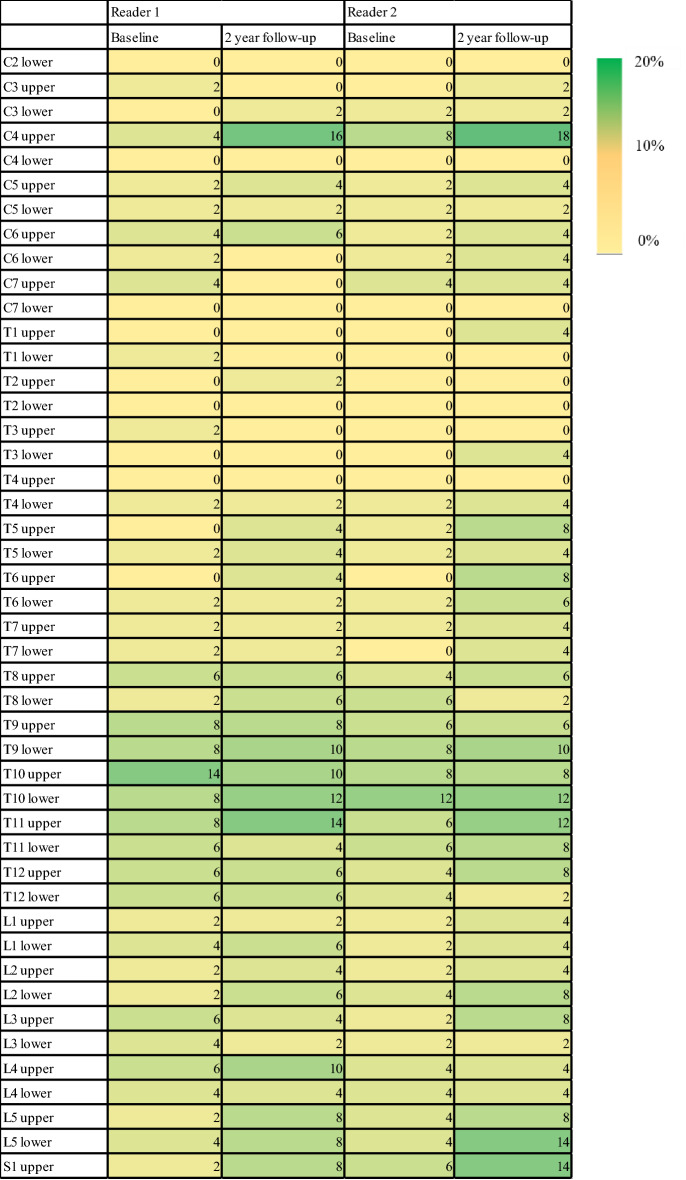
Distribution of syndesmophytes found on only the coronal view (lateral side) at baseline and at 2 year follow-up (number inside each cell is percentage).

Finally, we examined vertebral corners at which syndesmophytes were found only on the lateral side of the vertebral body (i.e., coronal view). Among 2300 vertebral corners (50 patients × 46 vertebral corners per patient), readers 1 and 2 identified 544 and 579 syndesmophytes at baseline, respectively. Among these, 72/544 (13.2%) and 66/579 (11.4%) vertebral corners had syndesmophytes only on the lateral side (right or left). Similar to the baseline CT findings, readers 1 and 2 identified 13.4% (98/734) and 15.2% (117/769) of vertebral corners as having only lateral side syndesmophytes at the 2-year follow-up. The distribution and percentage of syndesmophyte detected only in lateral side were described in Fig. 2.

## Discussion

The present study suggests a new method (sCTSS) for evaluating spinal structural damage associated with AS. This method was compared with the CTSS. Both methods were equally good at detecting meaningful spinal progression; however, the ICC for detecting changes in the syndesmophyte scores between baseline and 2-year follow-up was slightly better for the sCTSS.

Although detailed and fine subdivisions in the scoring methods for syndesmophytes can detect small amounts of progression, it can reduce inter-reader reliability. Also, although IDSs are well-defined in the lumbar spine, those in the cervical and thoracic spine are relatively narrow, making it difficult to discriminate score 1 from between 2 on the CTSS; this is particularly true in coronal plane images of the C spine in which the joint spaces are not parallel but tilted about 45°–60° (Supplementary Fig. 6). In AS patients, syndesmophytes grow vertically; therefore, discriminating grade 1 and 2 syndesmophyte growth using the CTSS is almost impossible in the C spine. The sCTSS method combines scores 1 and 2 of CTSS into a single score (score 1). This may increase the inter-reader reliability and explain why the ICC for detecting changes in the syndesmophyte score was better for the sCTSS than the CTSS.

A previous study reported that syndesmophytes were most common on the posterior lateral side of the vertebral column^9^. However, that study used three-dimensional reconstructed images of CT scans, which is uncommon in a clinical setting. Here, we evaluated the number and percentage of vertebral corners with syndesmophytes only on the lateral side; these syndesmophytes were present at 11.4–15.2% of vertebral corners. The time taken to evaluate the sCTSS for each whole spine image (15–20 min) was one-third to one-half of that taken to evaluate the CTSS (30–40 min). However, the detection rates for spinal progression were comparable. Although neglecting syndesmophytes on the lateral side may increase the risk of missing syndesmophyte progression slightly, the sCTSS can save much time, with a detection rate similar to that of the CTSS.

This study has several limitations. First, the number of enrolled patients were relatively small. Second, we compared the ICC for readers 1 and 2, even though the sCTSS has not been validated. Third, although the time taken for sCTSS was about half that taken for the CTSS, each whole spine CT still required 15 min. The CTSS was developed at 2018 and was based on a SIAS cohort^6,7^; no further research based on the CTSS has been published. Many clinical trials of axSpA (including AS) still evaluate radiographic progression using the mSASSS^4^, even though it has critical limitations^7^. The biggest hurdle to using the CTSS is the time taken and the complexity of the scoring system. The proposed sCTSS overcomes the limitations of the mSASSS, and has detection rates comparable with those of CTSS, but in half the time. Fourth, the sCTSS omits syndesmophyte on coronal view of CT (lateral side), it could miss syndesmophyte progression in lateral side of vertebral body. The previous study showed that posterolateral rim was the most common site of syndesmophyte involvement^9^. Actually the detection rate of syndesmophyte progression (change of CTSS or sCTSS over SDC) was decreased in reader 2, whereas increased in reader 1 between score based on CTSS and sCTSS. This may arise from the detection rate of syndesmphyte on lateral side, and which is the most critical limitation of sCTSS. The final limitation is that we did not compare the utility of sCTSS was with that of the mSASSS; this was because most AS patients in the present study did not have lateral view X-rays of the C and L spines at the same time when whole spine CT were taken.

In conclusion, the sCTSS detects radiographic progression of AS as well as CTSS, but in half the time and with a better ICC, making it a viable alternative to the CTSS.

## Supplementary Information

Supplementary Information.

## Data Availability

The datasets generated and/or analyzed in this study are available from the corresponding author upon reasonable request.

## Abbreviations

AS: Ankylosing spondylitis
CI: Confidence interval
CT: Computed tomography
CTSS: Computed tomography syndesmophyte score
ICC: Intraclass correlation coefficient
IDS: Intervertebral disc space
mSASSS: Modified Stoke Ankylosing Spondylitis Spinal Score
sCTSS: Simplified Computed Tomography Syndesmophyte Score
SDC: Smallest detectable change
TNFi: Tumor necrosis factor inhibitor
