# Supplementary material for: Proposal of simplified CT syndesmophyte score (sCTSS) and comparison with CTSS in patients with ankylosing spondylitis
Source: Sci Rep. 2023 Jan 23;13:1283. doi: 10.1038/s41598-023-28525-z (PMC9871040; doi:10.1038/s41598-023-28525-z)

Supplementary Table 1. Comparison of CTSS and sCTSS methods

|  | CTSS | sCTSS |
| --- | --- | --- |
| Spinal segment assessed |  |  |
| Cervical spine | Lower border of C2 to upper border of T1 | Lower border of C2 to upper border of T1 |
| Thoracic spine | Lower border of T1 to upper border of T12 | Lower border of T1 to upper border of T12 |
| Lumbar spine | Lower border of T12 to S1 | Lower border of T12 to S1 |
| Range of scoring system | 0–552 | 0–184 |
| Sites per vertebral endplate |  |  |
| Assessment at | Four quadrants (anterior, posterior, left lateral, right lateral) | Two quadrants (anterior, posterior) |
| Scoring grade |  |  |
| 0 | No syndesmophytes | No syndesmophytes |
| 1 | Syndesmophytes < 50% of IDS | Syndesmophytes, but not bridging |
| 2 | Syndesmophytes ≥ 50% of IDS but not bridging | Bridging syndesmophyte |
| 3 | Bridging syndesmophytes |  |
| Definition of syndesmophytes |  |  |
| New | Score 0 -> 1, 2, 3 | Score 0 -> 1, 2 |
| Growth | Score 1 -> 2, 3 or 2 -> 3 | Score 1 -> 2 |

Supplementary Figure 1. Example of scoring between CTSS and sCTSS


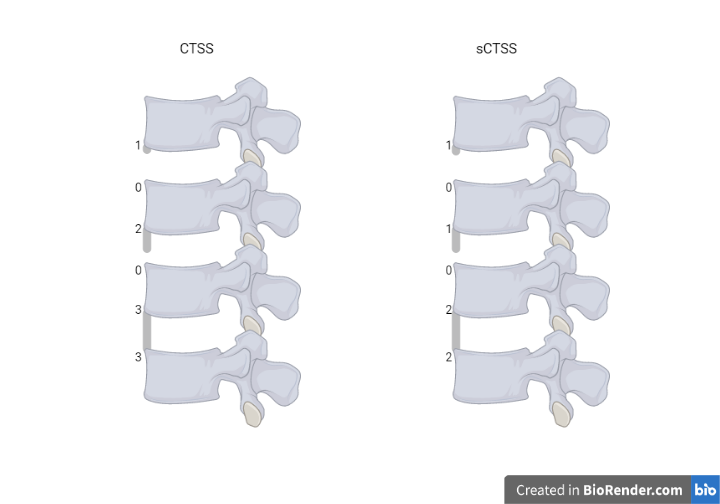


Supplementary Figure 2. Distribution of syndesmophytes at baseline and at the 2 year follow-up for CTSS (number inside each cell is percentage).

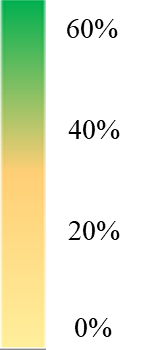


Supplementary Figure 3. Distribution of syndesmophytes at baseline and at the 2 year follow-up for sCTSS (number inside each cell is percentage).

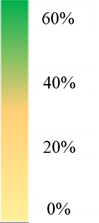


Supplementary Figure 4. The Bland-Altman plots for CTSS and sCTSS at (A) baseline, (B) 2 year follow up, (C) and change between baseline and 2 year follow up

**
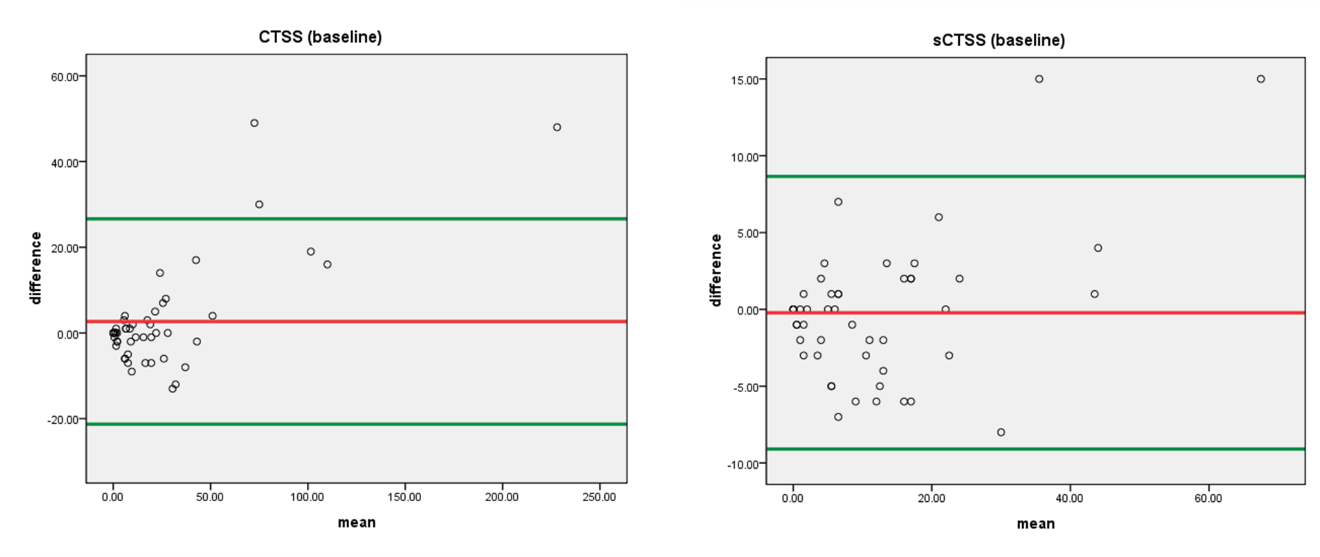
(A)**

**
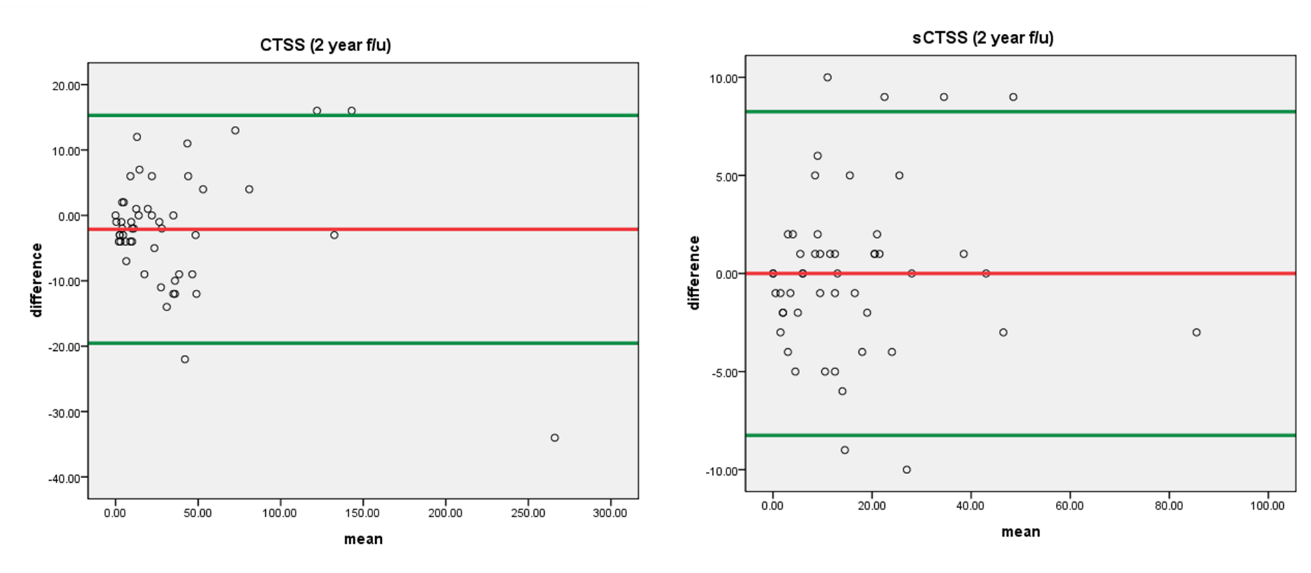
 (B)**

**(C)**

**
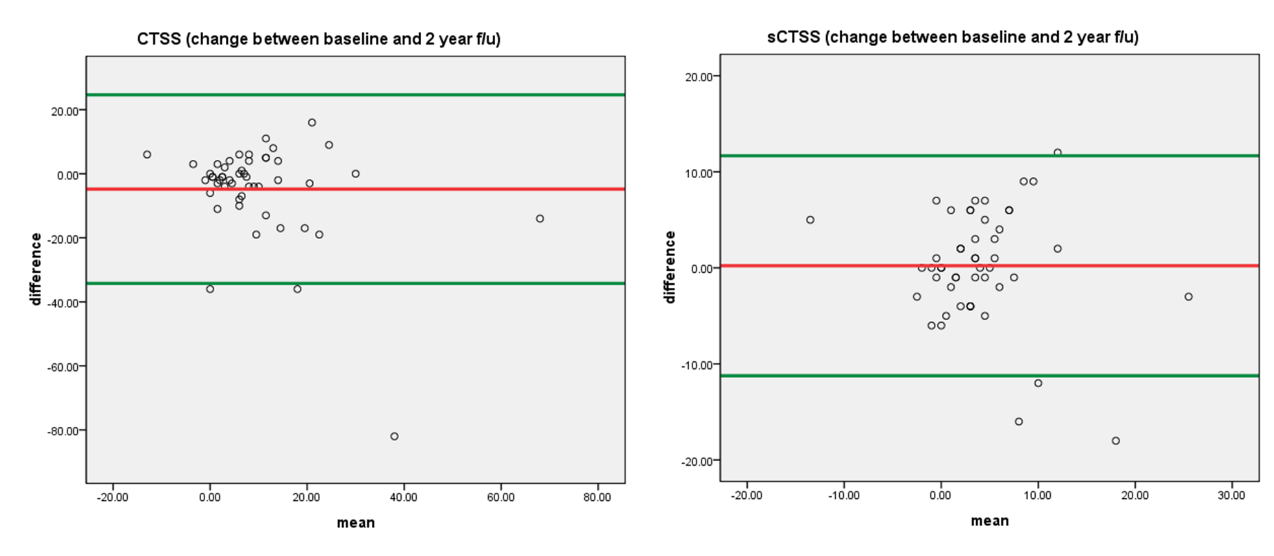
**

Supplementary Figure 5. Distribution of syndesmophytes progression or new syndesmophyte in CTSS and sCTSS (number inside each cell is percentage).

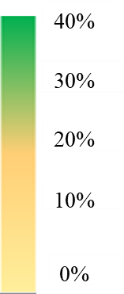


Supplementary Figure 6. Coronal view of the cervical spine. White arrow: syndesmophyte.


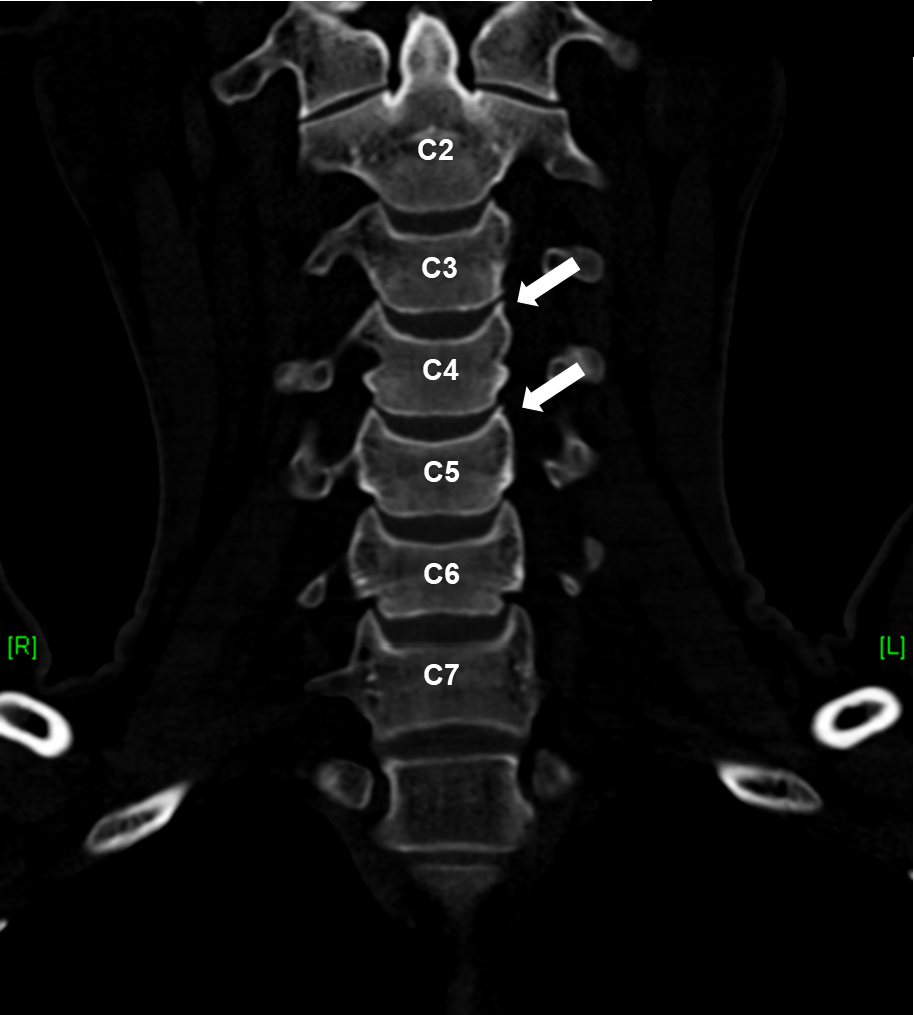

Supplement: Supplementary file 1 — Supplementary Information. [file 41598_2023_28525_MOESM1_ESM.docx]
